# Evolution of multiple sclerosis in France since the beginning of hepatitis B vaccination

**DOI:** 10.1007/s12026-014-8574-4

**Published:** 2014-11-14

**Authors:** Dominique Le Houézec

**Affiliations:** REVAHB (“Réseau Vaccin Hépatite B” in French), 32 rue du Clos Herbert, 14000 Caen, France

**Keywords:** Hepatitis B vaccine, Multiple sclerosis, Demyelinating disease, Pharmacovigilance, Vaccine adverse events

## Abstract

Since the implementation of the mass vaccination campaign against hepatitis B in France, the appearance of multiple sclerosis, sometimes occurring in the aftermath of vaccinations, led to the publication of epidemiological international studies. This was also justified by the sharp increase in the annual incidence of multiple sclerosis reported to the French health insurance in the mid-1990s. Almost 20 years later, a retrospective reflection can be sketched from these official data and also from the national pharmacovigilance agency. Statistical data from these latter sources seem to show a significant correlation between the number of hepatitis B vaccinations performed and the declaration to the pharmacovigilance of multiple sclerosis occurring between 1 and 2 years later. The application of the Hill’s criteria to these data indicates that the correlation between hepatitis B vaccine and multiple sclerosis may be causal.

## Introduction

The first doubts regarding vaccines as a possible cause or exacerbation of multiple sclerosis (MS) were formulated by Miller more than half century ago [1]. Hepatitis B (HB) vaccine has been the subject of greatest concern, especially in France where mass HB vaccine administration was performed in a short time. In 1992, the World Health Organization (WHO) recommended undertaking a universal HB vaccination of all young infants in order to eradicate the HB virus. WHO explained that the teenagers’ vaccination could also be used in addition to or instead of the vaccination of young children in low-endemic countries. In 1994, the French health authorities launched a national vaccination campaign of all pupils in the first year of secondary school. The following year, HB vaccine was added to the national immunization program for all young babies and preteenagers. This intensive campaign had quickly exceeded its expected targets by also encouraging the adult population to be mass-vaccinated, whereas the vaccination of the infants remained less significant. This resulted in an unprecedented “wave” of immunization in adults, with 20 million French individuals vaccinated against HB, concentrated in 4 years, from 1994 to 1997.

MS cases in some vaccinated adults were rapidly notified to the French national pharmacovigilance system (ANSM), triggering investigation by this agency. This inquiry, started in 1994, was therefore already underway when French media revealed possible occurrence of post-immunization MS in 1998. This year, French health authorities abruptly terminated routine school-based vaccination of preteens, and adult HB vaccination began to be less widespread.

Several epidemiological studies have been evaluating the correlation between HB vaccination and MS in adults for a decade. Most of these publications found the absence of a link [2–6] or a slightly increased risk, but not sufficiently significant on the statistical level [7–9]. However, different opinions have also been formulated. A study aiming at quantifying underreporting in Fourrier’s article [8] was conducted by D. Costagliola on request of the French pharmacovigilance. This unpublished study showed by the “capture–recapture” method that the real number of MS cases linked to HB vaccine was 2–2.5 higher than the officially registered number [10]. This additional calculation makes Fourrier’s publication [8] clearly significant. Another case–control epidemiological study was conducted to evaluate serious post-vaccination adverse events registered in the United States through a spontaneous reporting system in the VAERS database. Adults receiving HB immunization had significantly increased odds ratios (OR) for MS (OR 5.2; CI 1.9–20) in comparison with an age-, sex-, and vaccine year-matched unexposed tetanus-containing vaccine group [11]. A Hernan’s paper, based on a case–control study in the United Kingdom within the General Practice Research Database (GPRD), found an increased risk (OR 3.1; CI 1.5–6.3) of MS within the 3 years following HB immunization [12]. In the same way, a French study on demyelination in childhood [13] showed that Engérix B^®^ vaccine administration was associated with an increased trend of confirmed MS after 3 years (OR 2.77; CI 1.23–6.24).

On these grounds, we compared temporal HB vaccine dose distribution and MS occurrence in the French population, using the official data collected by the French pharmacovigilance system (ANSM) and the national health insurance (CNAM). The results confirmed, at the global population level, a significant correlation between the number of immunizations and both the number of MS cases declared to the pharmacovigilance system 1–2 years later and an overall increase in identified MS cases in the country.

## Materials and methods

### Databases

We compared data from two independent national databases: the National Health Service database (CNAM) [14] and the French pharmacovigilance system (ANSM) [15].

#### CNAM

The French general insurance provides each year the number of new cases of MS in which care is fully supported. These data are available online on the Web site of the CNAM [14]. The concerned population represents a very large majority of people covered by the healthcare system (83 % of the French population in 1996).

#### ANSM

This organization identifies spontaneous adverse event reports emerged in the aftermath of vaccinations since the beginning of the establishment of HB immunization (1981). The most common diseases reported were neurological damages of myelin, known under the generic term of demyelinating diseases. This condition is clinically called MS when at least two attacks of demyelination repeat themselves. When the neurological disorder remains single, without temporal or spatial diffusion, we speak of central nervous system demyelination.

The French pharmacovigilance is based on “spontaneous reporting” of adverse drug reactions. This allows the establishment of a possible relationship as well as the imputability to generate alerts. However, this system underestimates the real frequency of adverse reactions (1–10 % of severe side effects are reported) [16].

On the other hand, from 1997, the notification by REVAHB, the association of victims of HB vaccine, allowed the completion of these spontaneous reports of potential side effects. Since its inception, this association has been able to transmit more than 2,000 files of individuals who have experienced a neurological problem of post-vaccine demyelination. However, about a third of these files are not used by the French pharmacovigilance (classified as “not documented”) when the physician does not answer to the questionnaire which ANSM sends him for confirming the diagnosis. Of course, this rate of not documented files is an obvious factor of underreporting.

### Statistical analyses

We used the R statistical software to compute correlations and perform linear regressions.

## Results

### CNAM data analysis

The number of MS was very stable, about 2,500 new cases each year until 1993. The following years, and especially since 1996, a progressive increase in the number of new MS reported to the Health Insurance occurred. This figure increased to about 4,500 cases in 2003 and remains steady since.

**Fig. 1:**
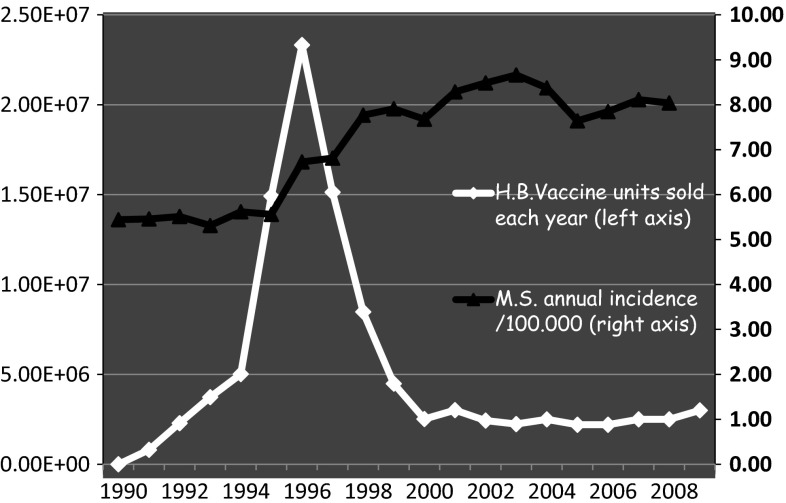
Evolution of annual incidence rate of MS supported by the French health insurance system (CNAM), comparison with annual sales of Hepatitis B (HB) vaccine in France (1990–2009)

The annual incidence was 5.3/10^5^ in 1993 and increased to 8.7/10^5^ insured people a decade later (Fig. 1), which translates into a 65 % increase in incidence over the 10-year period. These figures are consistent with epidemiological data published in this country. Indeed, the incidence of MS in France was estimated at around 4.3/10^5^ inhabitants in the years 1993–1997 from a representative sample of the Burgundy region [17]. It was reassessed by the same team at a rate between 7.6 and 8.8/10^5^ inhabitants for the period 2001–2007, from French CNAM data [18].

Epidemiological studies measuring prevalence of this disease provide an increase in the same magnitude. This figure was 40/10^5^ insured people in 1994, at the beginning of the mass vaccination campaign [19]. It increases rapidly until 95/10^5^ 12 years later [20].

### ANSM data analysis

**Fig. 2:**
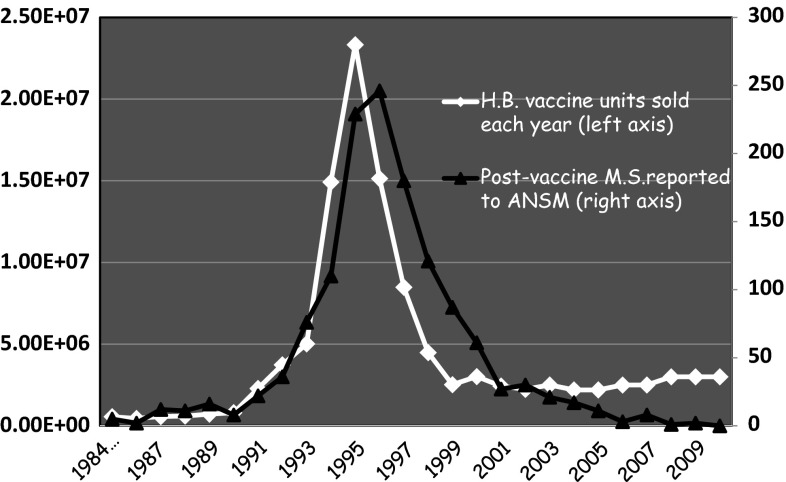
Sales of Hepatitis B (HB) vaccine every year in France, comparison with report of post-vaccine MS to the national pharmacovigilance agency (ANSM) (1984–2010)

Since the beginning of practicing HB vaccination in France until December 31, 2010, ANSM has registered 1,650 demyelinating diseases including 1,418 MS. These data are available online on the Web site of ANSM in the French national commission for pharmacovigilance of September 27, 2011 [15]. When you draw a distribution curve of MS reported each year to ANSM in the aftermath of a vaccine injection, we see that this distribution is neither linear nor regular, far from it (Fig. 2). There is a huge peak of reported MS culminating in the years 1995 (229 reports) and 1996 (246 reports). This peak of post-vaccine neurological disorders during the period 1994–1998 corresponds, with an interval of one year, to the beginning of the campaign and intense promotion of the HB vaccination in France (culminating in the year 1995 with about 23 million vaccine doses sold).

We studied the correlation between MS data (*Y*) and vaccinations data (*X*). This correlation is high and maximum (0.9365863) between the number of vaccines sold at *t* time (called *Xt*) and the number of MS occurring the following year, *t* + 1 (called *Yt* + 1). There is also a high correlation (0.7350417) between vaccines sold at t time (*Xt*) and the number of reported MS 2 years later (called *Yt* + 2).

If we model this relationship in a linear fashion without constant (since in the absence of vaccination there are no MS cases registered by pharmacovigilance), the best model is one where the coefficient of determination adjusted *R* ^2^ is the highest (i.e., = 0.9497).

This model is defined by the relation: *Yt* + 2 = *ß*1*Xt* + *ß*2*Xt* + 1 + *ß*3*Xt* + 2

The series of sold vaccines at t time (*Xt*) and 1 year later (*Xt* + 1) have a significant influence (*p* = 0.00106 for *Xt* and 0.02491 for *Xt* + 1) on the number of reported MS at *t* + 2 years (*Yt* + 2), i.e., 2 years later. But we cannot say whether the number of vaccines sold in year *t* + 2 (*Xt* + 2) has a significant influence (*p* = 0.07014). Graphically, this relation is also the model that best fits the peak of reported MS to ANSM.

It is difficult to adjust the MS data after year 2002. There is then a notable difference between the theoretical series (models) and the actual series. This can be explained by the fact that the number of vaccinations mentioned by ANSM became less precise figures, rounded and approximate. In addition, since 1999, the immunization target has been focused on young children. Adult vaccination has become uncommon, reserved only for high-risk groups. Finally, the number of MS reported to pharmacovigilance has arguably become more and more underestimated over the years. The problem of the emergence of post-vaccine MS had been widely publicized in the years 1996–1999. Thereafter, over the years, this problem has been trivialized or forgotten. Since this period, underreporting became more important. People who have been victims of adverse events have not necessarily reminded the physician of the injection of a HB vaccine some weeks or months before.

## Discussion

Are we able to establish a relation between these results and the Hill’s criteria [21]? Is there a causal relationship between the HB vaccination and the incidence of MS in France? The Hill’s criteria for causation include nine items detailed in Table 1. We will detail now the most important criteria in the text, the other being a simple bibliographic reference mentioned in this table.

**Table 1:** Study of Hill’s criteria

Criteria	Results	Comments
1. Strength of the association (appropriate statistical tests)	Yes	See text
2. Consistency of the observed association	Yes	See text
3. Specificity of the association	No	Not applicable to diseases such as MS
4. Temporal relationship of the association	Yes	See text
5. Biological gradient or dose–response curve	No	Acceptable when “the mere presence of the factor can trigger the effect” [21]
6. Biological plausibility	Yes	See text
7. Coherence with the current knowledge	Yes	Possible role of the vaccine aluminum adjuvant [22]
8. Experimental or semi-experimental evidence	Yes	Inducing experimental allergic encephalomyelitis [23, 24]
9. Analogy with similar evidence	Yes	Occurrence of Guillain–Barré syndrome after HB vaccine [25]

The current study satisfies the first criterion. The association is highly statistically significant between reported MS (*Yt* + 2) to pharmacovigilance and the series of HB vaccines that were sold 1 and 2 years before (*p* < 0.01 for sold vaccines 2 years before (*Xt*) and *p* < 0.05 for sold vaccines 1 year before (*Xt* + 1); adjusted *R* ^2^ = 0.9497). Although it is possible to demonstrate here a statistical relationship between the number of sold vaccines and MS reported to the pharmacovigilance, it is not enough to affirm an absolute causality. This is a strong signal that requires further epidemiological studies.

The positive and statistically significant correlation between HB vaccine exposure and reported MS incidence is consistently observed in different places, circumstances, and times (criterion 2).

First, this result is consistent with the Hernan’s case–control study [12] that found in the British population an increased risk of MS (OR 3.1; CI 1.5–6.3) in the 3 years following HB vaccination. Moreover, in this same study, the risk was greater when the last immunization took place within the second or third years before first symptoms of MS (OR 4.1; CI 1.3–13.6).

The results of the case–control study by Geier [11] in USA are also consistent with the French pharmacovigilance data. There is a very significant change in the risk of developing MS after HB vaccine in adults in the VAERS database (OR 5.2, *p* < 0.0003; CI 1.9–20).

The Costagiola’s study [10] found underreporting of post-vaccine reported MS during the observation period (1994–1996) of an epidemiological study requested by French pharmacovigilance [9]. The combination of these two studies suggests a real number of cases significantly higher (RR = 1.66) than the expected number of MS during the 3 years of the collection.

Most publications where there is no link between HB vaccination and the onset of MS [2–5] received grants from pharmaceutical industry. Other criticism that can be raised for some of these negative case–control studies is the limited period (2–24 months) of their survey [4, 7–9]. Moreover, the Hernan’s publication [12] shows also a negative result (OR 1.8; CI 0.5–6.3) for a period of 1 year and becomes significant between 2 and 3 years of follow-up after HB immunization.

The case–control study nested in the Nurses’ Health by Asherio [4] presents several biases. The vaccination status was obtained retrospectively like the date of first symptoms of the disease assessed by questionnaires. This process may cause selection bias leading to a downwardly biased OR as the specific (nurses) selected population [26].

At last, a meta-analysis [27], based on six epidemiological case–control studies [4–7, 11, 12], did not find significant change in the risk of developing MS after HB vaccine in adults (OR 0.92; CI 0.84–1.004). This paper can also be criticized. Strangely, the statistical computing of this meta-analysis attributes a non-significant value to the Hernan’ study [12], with an OR 1 (CI 0.5–2.1) by using the cases’ date of diagnosis as the index date instead of the date of first symptoms as the author does. But as Hernan wrote [12], “the use of dates that are posterior to the true date of first symptoms may cause a downward bias of the OR for acute exposures such as vaccinations”. In addition, the most significant study by Geier [11] is removed, being regarded as a “source of heterogeneity”. So, withdrawal of a positive study and changing the result of another one more easily allows a negative outcome.

Generally speaking, we know that a low risk of adverse post-vaccination cannot be demonstrated by studies of low statistical power with small numbers of exposed people. Therefore, results in a population of over 20 million vaccinated people should attract attention and require further epidemiologic studies. Moreover, studies with a short period of post-vaccination monitoring are inadequate because they do not take into account the long biopersistence of immunostimulatory vaccine compounds (such as aluminum hydroxide) in the body. In this, vaccines derogate from the rule generally used for side effects of drugs.

The temporal relationship (criterion 4) clearly exists here. The annual incidence of MS recorded by the French insurance was stable about 5.5/10^5^ until 1995. It rose sharply in 1996 to stabilize around 8/10^5^ from 1998. But this sharp increase (65 %) closely follows a major peak in the number of vaccines sold between 1995 and 1997 in France (Fig. 1). The number of MS occurring in the aftermath of a HB vaccination reported to the French pharmacovigilance almost draws the same peak with a delay between 1 and 2 years (Fig. 2). Moreover, some papers report observations of MS relapses triggered by repeated injections of HB vaccine [28, 29].

The official explanations of the increase in this incidence are twofold, first a better screening of MS whose diagnosis has been made easier and faster by using radiological data provided by MRI. This is a dubious explanation. This new radiological technique has begun to develop gradually in French hospitals in 1990 and thus before the obvious increase in the recruitment of MS by French national insurance (1996). Otherwise, if this earlier diagnosis was really so important in the increased incidence of MS, we should have observed in France a decrease in the average age of newly diagnosed cases. And this rejuvenation was not observed [30].

The second factor involves the change in treatment protocol of this period with the introduction of treatment with interferon-beta in 1995, an innovative and very expensive drug that prompted physicians to quickly seek a total care by French health insurance. In 2004, the emergence of a new drug (glatiramer), indicated for the most common form of MS (relapsing–remitting), has not been followed by an increase in cases registered by CNAM that year and the following. The incidence remained the same. This explanation cannot alone explain a so rapid and significant increase (65 % over 4 years) in the incidence of a disease like MS.

A third factor must be considered in such a sudden increase in MS incidence. So the changing of an environmental etiological factor must be taken into account seriously. This therefore appears to be the case for the question of the potential role of HB vaccination carried out in France for a short time and in a massive way, about 20 million people concentrated in 4 years. It is interesting to compare these figures with those countries where routine vaccination has not been recommended. In Norway, the incidence of MS is higher than in France in the early 1990 s (8.7/10^5^ between 1990 and 1995). Then, it decreases slightly in subsequent years (7.2/10^5^ from 1996 to 2000) [31]. In the county of Värmland (Sweden), the incidence of MS has remained similar (6.4/10^5^) during the periods 1991–1995 and 1996–2000 [32].

Specificity (criterion 3) is likely for a very specific population at a specific site and disease. This is not applicable to diseases such as MS. Genetic risk (HLA-DR2) and environmental factors (vitamin D insufficiency) or infectious factors (Epstein–Barr virus, endogenous retroviruses) are clearly involved in the occurrence of MS although its etiology and pathophysiology are not completely understood. These other environmental and genetic factors may have contributed to the raise in MS incidence and should be mentioned.

Biological plausibility (criterion 6): A plausible mechanism between cause and effect is helpful. Are there explanations regarding plausible mechanisms by which vaccines and particularly this vaccine may induce harm? This issue has been extensively studied in recent years. Various aspects of the causal and temporal interactions between vaccines and autoimmune phenomena are known, as well as the possible mechanisms by which different components of vaccines might induce autoimmunity [33]. A first hypothesis could be the similarity between the protein S (used in the vaccine against HB) and some myelin proteins such as PLP (proteolipid proteins) [34]. Another interesting track would be contamination by minor HB virus polymerase proteins. And we know that HB virus polymerase shares significant amino acid similarities with the human MBP (myelin basic protein) [35]. This process is called molecular mimicry: a foreign antigen that shares sequence or structural similarities with self-antigens.

Another runway about biological plausibility is to take into account the metabolism of vaccine adjuvants in the human body. The long-term persistence of aluminum adjuvant at the site of vaccine injection is now well established [36]. Furthermore, transferring of aluminum particles from muscle to brain is demonstrated in animals [37]. A new syndrome entitled ASIA, “Autoimmune (Auto-inflammatory) Syndrome Induced by Adjuvants”, was recently described, grouping four similar illnesses [38]. These diseases (siliconosis, the Gulf war syndrome, the macrophagic myofasciitis syndrome and post-vaccination phenomena) were linked with previous exposure to an immune adjuvant (silicone, aluminum salts). In another publication, the same authors found common clinical characteristics of the ASIA criteria among 93 patients diagnosed with immune-mediated conditions post-HB vaccination, suggesting a common denominator in these diseases [39].

## Conclusions

The figures available in France thus show a definite statistical signal in favor of a causal link between the HB vaccine event and the apparition of MS with a maximum correlation in the 2 years following immunization. The impact of other factors (new use of MRI, beginning of interferon-beta) is probably associated. The weakness of this study is its retrospective nature and therefore subject to bias of notoriety. Its strength is that it is based on indisputable official data on large numbers and during about 12 years. The appearance of a spectacular “vaccine wave” in France has remained the only one in its kind. The intensive lobbying carried out in the years 1994–1997 led to concentrate as many vaccinated people as possible in the shortest period of time. This particularity is perhaps the explanation of the emergence of the problem of post-vaccine MS, especially recorded in this country. The low overall frequency of this adverse effect, not measurable in most epidemiological studies, here becomes more obvious because of a kind of involuntary very large scale experiment carried out on a third of the French population. All this is expected to require further epidemiological studies, particularly from the French health insurance data. Indeed, CNAM has information on millions of insured persons for many years that would be usable if we could more easily access it.

## Abbreviations

ANSM: Agence nationale de sécurité du médicament et des produits de santé in French
CI: Confidence interval
CNAM: Caisse nationale d’assurance maladie in French
GPRD: General Practice Research Database
HB: Hepatitis B
MBP: Myelin basic protein
MRI: Magnetic resonance imaging
MS: Multiple sclerosis
OR: Odds ratio
*p*: *p* value
PLP: Proteolipid proteins
*R*^2^: Coefficient of determination R-squared
RR: Relative risk
REVAHB: Réseau vaccin hépatite B in French
VAERS: Vaccine Adverse Events Reporting System
WHO: World Health Organization
